# The dynamics underlying pseudo-plateau bursting in a pituitary cell model

**DOI:** 10.1186/2190-8567-1-12

**Published:** 2011-11-08

**Authors:** Wondimu Teka, Joël Tabak, Theodore Vo, Martin Wechselberger, Richard Bertram

**Affiliations:** 1 Department of Mathematics; Florida State University, Tallahassee, FL, USA; 2 Department of Biological Science; Florida State University, Tallahassee, FL, USA; 3 School of Mathematics and Statistics; University of Sydney, Sydney, NSW, Australia; 4 Department of Mathematics, and Programs in Neuroscience and Molecular Biophysics; Florida State University, Tallahassee, FL, USA

**Keywords:** Bursting, Mixed mode oscillations, Folded node singularity, Canards, Mathematical model

## Abstract

Pituitary cells of the anterior pituitary gland secrete hormones in response to patterns of electrical activity. Several types of pituitary cells produce short bursts of electrical activity which are more effective than single spikes in evoking hormone release. These bursts, called pseudo-plateau bursts, are unlike bursts studied mathematically in neurons (plateau bursting) and the standard fast-slow analysis used for plateau bursting is of limited use. Using an alternative fast-slow analysis, with one fast and two slow variables, we show that pseudo-plateau bursting is a canard-induced mixed mode oscillation. Using this technique, it is possible to determine the region of parameter space where bursting occurs as well as salient properties of the burst such as the number of spikes in the burst. The information gained from this one-fast/two-slow decomposition complements the information obtained from a two-fast/one-slow decomposition.

## 1 Introduction

Bursting is a common pattern of electrical activity in excitable cells such as neurons and many endocrine cells. Bursting oscillations are characterized by the alternation between periods of fast spiking (the active phase) and quiescent periods (the silent phase), and accompanied by slow variations in one or more slowly changing variables, such as the intracellular calcium concentration. Bursts are often more efficient than periodic spiking in evoking the release of neurotransmitter or hormone [1-3].

**Figure 1:**
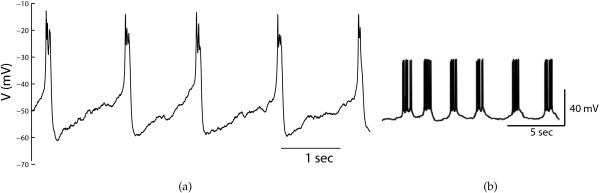
**(a) Pseudo-plateau bursting in a GH4 pituitary cell line**. (b) Plateau bursting in a neonatal CA3 hippocampal principal neuron. Reprinted with permission from [14].

The endocrine cells of the anterior pituitary gland display bursting patterns with small spikes arising from a depolarized voltage [2-5]. Similar patterns have been observed in single pancreatic *β*-cells isolated from islets [6-8]. Figure 1(a) shows a representative example from a GH4 pituitary cell. Several mathematical models have been developed for this bursting type [5,8-10]. Prior analysis showed that the dynamic mechanism for this type of bursting, called pseudo-plateau bursting, is significantly different from that of square-wave bursting (also called plateau bursting) which is common in neurons [11-13]. Yet this analysis did not determine the possible number of spikes that occur during the active phase of the burst. The goal of this paper is to understand the dynamics underlying pseudo-plateau bursting, with a focus on the origin of the spikes that occur during the active phase of the oscillation.

Minimal models for pseudo-plateau bursting can be written as

(1.1) $$\varepsilon_{1}\dot{V}=f\left(V,n,c\right)$$

(1.2) ṅ=g(V,n)

(1.3) $$ċ=\varepsilon_{2}h\left(V,c\right)$$

where *V *is the membrane potential, *n *is the fraction of activated delayed rectifier K^+ ^channels, and *c *is the cytosolic free Ca^2+ ^concentration. The velocity functions are nonlinear, and ε_1 _and ε_2 _are parameters that may be small.

The variables *V*, *n*, and *c *vary on different time scales (for details, see Section 2). By taking advantage of time-scale separation, the system can be divided into fast and slow subsystems. In the standard fast/slow analysis one considers *ε*_2 _≈ 0, so that *V *and *n *form the fast subsystem and *c *represents the slow subsystem. One then studies the dynamics of the fast subsystem with the slow variable treated as a slowly varying parameter [12,15-18]. This approach has been very successful for understanding plateau bursting, such as occurs in pancreatic islets [19], pre-Bötzinger neurons of the brain stem [20], trigeminal motoneurons [21] or neonatal CA3 hippocampal principal neurons [14], Figure 1(b). It has also been useful in understanding aspects of pseudo-plateau bursting such as resetting properties [11], how fast subsystem manifolds affect burst termination [17], and how parameter changes convert the system from plateau to pseudo-plateau bursting [12].

An alternate approach, which we use here, is to consider *ε*_1 _≈ 0, so that *V *is the sole fast variable and *n *and *c *form the slow subsystem. With this approach, we show that the active phase of spiking arises naturally through a canard mechanism, due to the existence of a folded node singularity [22-25]. Also, the transition from continuous spiking to bursting is easily explained, as is the change in the number of spikes per burst with variation of conductance parameters. Thus, the one-fast/two-slow variable analysis provides information that is not available from the standard two-fast/one-slow variable analysis in the case of pseudo-plateau bursting.

## 2 The mathematical model

We use a model of the pituitary lactotroph, which produces pseudo-plateau bursting over a range of parameter values [10]. To achieve a minimal form, we use the model without A-type K^+ ^current (*I_A_*). It includes three variables: *V *(membrane potential), *n *(fraction of activated delayed rectifier K^+ ^channels), and *c *(cytosolic free Ca^2+ ^concentration). The equations are:

(2.1) $$C_{m}\frac{dV}{dt}=-\left(I_{Ca}+I_{K}+I_{K\left(Ca\right)}+I_{BK}\right)$$

(2.2) $$\frac{dn}{dt}=\frac{\left(n_{\infty }\left(V\right)-n\right)}{\tau_{n}}$$

(2.3) $$\frac{d\text{c}}{dt}=-f_{c}\left(\alpha I_{Ca}+k_{c}\;\text{c}\right)$$

where *I_Ca _*is an inward Ca^2+ ^current, *I_K _*is an outward delayed rectifying K^+ ^current, *I*_*K*(*Ca*) _is a small-conductance Ca^2+^-activated K^+ ^current, and *I_BK _*is a fast-activating large-conductance BK-type K^+ ^current. The currents in the equations above are:

(2.4) $$I_{Ca}=g_{Ca}m_{\infty }\left(V\right)\left(V-V_{Ca}\right)$$

(2.5) $$I_{K}=g_{K}n\left(V-V_{K}\right)$$

(2.6) $$I_{K\left(Ca\right)}=g_{K\left(Ca\right)}s_{\infty }\left(c\right)\left(V-V_{K}\right)$$

(2.7) $$I_{BK}=g_{BK}b_{\infty }\left(V\right)\left(V-V_{K}\right).$$

The steady state activation functions are given by:

(2.8) $$m_{\infty }\left(V\right)={\left(1+\exp\left(\frac{v_{m}-V}{s_{m}}\right)\right)}^{-1}$$

(2.9) $$n_{\infty }\left(V\right)={\left(1+\exp\left(\frac{v_{n}-V}{s_{n}}\right)\right)}^{-1}$$

(2.10) $$s_{\infty }\left(c\right)=\frac{c^{2}}{c^{2}+K_{d}^{2}}$$

(2.11) $$b_{\infty }\left(V\right)={\left(1+\exp\left(\frac{v_{b}-V}{s_{b}}\right)\right)}^{-1}.$$

Default parameter values are given in Table 1.

**Table 1:** Parameter values for the lactotroph model.

Parameter	Value	Description
*C_m _*	5 pF	Membrane capacitance of the cell
*g_Ca _*	2 nS	Maximum conductance of Ca^2+ ^channels
*V_Ca _*	50 mV	Reversal potential for Ca^2+^
*ν_m_*	-20 mV	Voltage value at midpoint of *m*_∞_
*s_m_*	12 mV	Slope parameter of *m*_∞_
*g_K_*	4 nS	Maximum conductance of K^+ ^channels
*V_K _*	-75 mV	Reversal potential for K^+^
*ν_n_*	-5 mV	Voltage value at midpoint of *n*_∞_
*s_n _*	10 mV	Slope parameter of *n*_∞_
*τ_n_*	43 ms	Time constant of *n*
*g*_*K*(*Ca*)_	1.7 nS	Maximum conductance of K(Ca) channels
*K_d_*	0.5 *μ*M	*c *at midpoint of *s*_∞_
*g_BK_*	0.4 nS	Maximum conductance of BK-type K^+ ^channels
*ν_b_*	-20 mV	Voltage value at midpoint of *f*_∞_
*s_b_*	5.6 mV	Slope parameter of *f*_∞_
*f_c _*	0.01	Fraction of free Ca^2+ ^ions in cytoplasm
*α*	0.0015 *μ*M fC^-1^	Conversion from charge to concentration
*k_c_*	0.16 ms^-1^	Rate of Ca^2+ ^extrusion

The variables *V*, *n *and *c *vary on different time scales. The time constant of *V *is given by *τ_V _*= *C_m_*/*g_Total_*, where *g*_*Total *_= *g_K_n *+ *g_BK_b*_∞_(*V*) + *g_Ca_m*_∞_(*V*) + *g*_*K*(*Ca*)_*s*_∞_(*c*). During a bursting oscillation, the minimum of *g_Total _*is 0.483 pS and the maximum is 3 pS. Hence, $\frac{C_{m}}{maxg_{Total}}\leq \tau_{V}\leq \frac{C_{m}}{ming_{Total}}$, or 1.7 ms ≤ *τ_V _*≤ 10.4 ms, for *C_m _*= 5 pF, a typical capacitance value for lactotrophs. The time constant for *n *is *τ_n _*= 43 ms. For the variable *c*, the time constant is $\frac{1}{f_{c}k_{c}}=\frac{1}{\left(0.01\right)\;\left(0.16\right)}$ ms = 625 ms. Thus, *n *and *c *change more slowly than *V*. This time scale separation between *V *and (*c*, *n*) can be accentuated when *C_m _*is made smaller than the default 5 pF, i.e., when *C_m _*→ 0, *τ_V _*gets smaller and *V *varies much faster. Thus, we can view the capacitance *C_m _*as a representative of the dimensionless singular perturbation parameter *ε*_1 _in this model (Eq. 1.1).

All numerical simulations and bifurcation diagrams (both one- and two-parameter) are constructed using the XPPAUT software package [26], using the Runge-Kutta integration method, and computer codes can be downloaded from the following website: http://www.math.fsu.edu/~bertram/software/pituitary. The surface in Figure 9 was constructed using the AUTO software package [27]. All graphics were produced with the software package MATLAB.

## 3 Geometric Singular Perturbation Theory

### The reduced system

We consider the full system (Eqs. (2.1)-(2.3)) as having one fast variable *V *and two slower variables *n *and *c*. The time-scale separation can be accentuated by decreasing the singular perturbation parameter *C_m_*. This facilitates analysis of the system dynamics [28]. In the limit *C_m _*→ 0, the trajectories of the system lie on a 2-D surface called the critical manifold. If we define the right hand side of Eq. (2.1) by

(3.1) $$f\left(V,c,n\right)=-\left(I_{Ca}+I_{K}+I_{K\left(Ca\right)}+I_{BK}\right)$$

then the critical manifold is the surface S satisfying

(3.2) $$S\equiv \left\{\left(V,c,n\right)\in {\mathbb{R}}^{3}:f\left(V,c,n\right)=0\right\}.$$

The equation *f*(*V*, *c*, *n*) = 0 can be solved in explicit form for *n *as

(3.3) $$n=n\left(c,V\right)=-\frac{1}{g_{K}}\left[g_{Ca}m_{\infty }\left(V\right)\frac{\left(V-V_{Ca}\right)}{\left(V-V_{K}\right)}+g_{K\left(Ca\right)}s_{\infty }\left(c\right)+g_{BK}b_{\infty }\left(V\right)\right].$$

**Figure 2:**
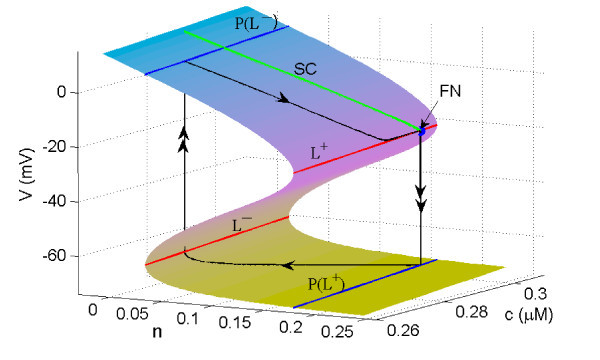
**The critical manifold and fold curves with their projections for *g_K _*= 4 nS and *g_BK _*= 0.4 nS**. The curves L^- ^and L^+ ^are the lower and upper fold curves, respectively. P(L^-^) and P(L^+^) are the projections of L^- ^and L^+ ^onto the upper and lower sheets of the critical manifold, respectively. FN is a folded node singularity, and SC (green curve) is the strong canard. The singular periodic orbit (black curve) is superimposed on the critical manifold.

The critical manifold (3.3) is a folded surface (Figure 2) that consists of three sheets separated by two fold curves (L^- ^and L^+^). The lower and upper sheets are attracting ($\frac{\partial f}{\partial V}<0$) and the middle sheet is repelling ($\frac{\partial f}{\partial V}>0$). The lower (L^-^) and upper (L^+^) fold curves are given by

(3.4) $${\text{L}}^{\pm }\equiv \left\{\left(V,c,n\right)\in {\mathbb{R}}^{3}:f\left(V,c,n\right)=0\text{ and }\frac{\partial f}{\partial V}\left(V,c,n\right)=0\right\}.$$

This yields two constant *V *values and two equations for *n *in the form of *n *= *n*(*c*). Thus, the fold curves (L^±^) are (*V*^±^, *c*, *n*^± ^(*c*)) where *V*^- ^and *V*^+ ^are constant *V *values. The curve L^+ ^is projected vertically (along the fast variable *V*) onto the lower sheet to obtain the projection curve P(L^+^), and similarly for the (L^-^) projection onto the upper sheet. Figure 2 shows the critical manifold, the fold curves and the projections of the fold curves.

The reduced flow (when *C_m _*→ 0) is described by (3.3), the differential equation for *c *(Eq. (2.3)), and a differential equation for *V *which can be obtained by differentiating *f*(*V*, *c*, *n*) = 0 with respect to time. That is,

(3.5) $$-\frac{\partial f}{\partial V}\frac{dV}{dt}=\frac{\partial f}{\partial c}\frac{dc}{dt}+\frac{\partial f}{\partial n}\frac{dn}{dt}$$

where *n *satisfies Eq. (3.3), and ,  satisfy Eqs. (2.2), (2.3). The two differential equations for the reduced system are thus

(3.6) $$-\frac{\partial f}{\partial V}\frac{dV}{dt}=\left(\right)-f_{c}(\alpha I_{Ca}+k_{c}\;\text{c})))\frac{\partial f}{\partial c}+\left(\frac{\left(n_{\infty }\left(V\right)-n\right)}{\tau_{n}}\right)\frac{\partial f}{\partial n}$$

(3.7) $$\frac{d\text{c}}{dt}=-f_{c}\left(\alpha I_{Ca}+k_{c}\;\text{c}\right).$$

Since $\frac{\partial f}{\partial V}=0$ on *L*^±^, the reduced system is singular along the fold curves. The system can be desingularized by rescaling time with $\tau =-{\left(\frac{\partial f}{\partial V}\right)}^{-1}t$. The desingularized system is then

(3.8) $$\frac{dV}{d\tau }=\left(\right)-f_{c}(\alpha I_{Ca}+k_{c}\;\text{c})))\frac{\partial f}{\partial c}+\left(\frac{\left(n_{\infty }\left(V\right)-n\right)}{\tau_{n}}\right)\frac{\partial f}{\partial n}$$

(3.9) $$\frac{d\text{c}}{d\tau }=f_{c}\left(\alpha I_{Ca}+k_{c}\;\text{c}\right)\frac{\partial f}{\partial V}.$$

Defining

(3.10) $$F\left(V,c,n\right)=\left(\right)-f_{c}\left(\alpha I_{Ca}+k_{c}\;\text{c}\right)))\frac{\partial f}{\partial c}+\left(\frac{\left(n_{\infty }\left(V\right)-n\right)}{\tau_{n}}\right)\frac{\partial f}{\partial n},$$

we have the desingularized system

(3.11) $$\frac{dV}{d\tau }=F\left(V,c,n\right)$$

(3.12) $$\frac{d\text{c}}{d\tau }=f_{c}\left(\alpha I_{Ca}+k_{c}\;\text{c}\right)\frac{\partial f}{\partial V}.$$

The desingularized system describes the flow on the critical manifold. Because of the time rescaling, the flow on the middle sheet, where $\frac{\partial f}{\partial V}>0$, must be reversed to obtain the equivalent reduced flow.

### FoLded singuLarities and canards

Equilibria of the desingularized system are classified as ordinary singularities and folded singularities. An ordinary singularity is an equilibrium of Eqs. (2.1)-(2.3) and satisfies

(3.13) f(V,c,n)=0

(3.14) $$n=n_{\infty }\left(V\right)$$

(3.15) $$c=-\frac{\alpha I_{Ca}}{k_{c}}.$$

A folded singularity lies on a fold curve (L^+ ^or L^-^), and satisfies:

(3.16) f(V,c,n)=0

(3.17) F(V,c,n)=0

(3.18) $$\frac{\partial f}{\partial V}=0.$$

A folded singularity is classified as a folded node if the eigenvalues are real and have the same sign, a folded saddle if the eigenvalues are real and have opposite signs, or a folded focus if the eigenvalues are complex [22,23,25,29]. For parameter values used in Figure 2, the system has a folded node (with negative eigenvalues) on L^+ ^(FN, blue point, in Figure 2), and a folded focus on L^-^(not shown).

**Figure 3:**
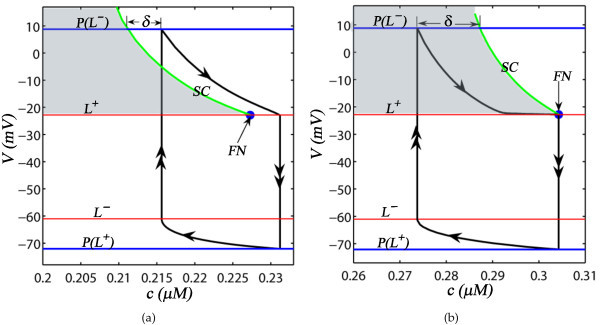
**The critical manifold is projected onto the (c, V)-plane for (a) *g_K _*= 5.1 nS, *g_BK _*= 0.4 nS and (b) *g_K _*= 4 nS, *g_BK _*= 0.4 nS**. L^- ^and L^+ ^are the lower and upper fold curves, respectively. P(L^-^) and P(L^+^) are the projections of L^- ^and L^+^. The shaded regions are singular funnels which are delimited by the curves *L*^+ ^and the strong canards (SC, green curves). The singular periodic orbits (black curves with arrows) are superimposed. FN is a folded node singularity. *δ *< 0 in panel (a) and *δ *> 0 in panel (b).

There are an infinite number of singular trajectories on the top sheet that pass through the folded node (FN). These are called singular canards [22]. The singular canard that enters the FN in the direction of the strong eigenvector is called the strong canard (SC, green curve, in Figure 2). This curve and the fold curve L^+ ^delimit the singular funnel that consists of all initial conditions whose trajectories for the reduced system pass through the folded node. The singular funnel and key curves are projected onto the (c, V)-plane in Figure 3. The different panels are obtained with different values of the parameter *g_K_*.

### Singular periodic orbits, relaxation oscillations, and mixed mode oscillations

A singular periodic orbit (Figure 2, black curve with arrows) can be constructed by solving the desingularized system for the flow on the top and bottom sheets of the critical manifold, and then projecting the trajectory from one sheet to the other along fast fibers when the trajectory reaches a fold curve. The singular periodic orbit is the closed curve constructed in this way. This process was discussed in detail in [22,28,30]. Briefly, the trajectory moves along the bottom sheet until L^- ^is reached. At this point the reduced flow is singular ($\frac{\partial f}{\partial V}=0$). The quasi-steady state assumption *f*(*V*, *c*, *n*) = 0 is no longer valid and there is a rapid motion away from the fold curve L^-^. This rapid motion is seen as vertical movement to the top sheet (the dynamics are governed by the layer problem, see [22,28]). The trajectory moves to a point on P(L^-^) and from there is once again governed by the desingularized equations, moving along the top sheet until L^+ ^is reached. The fast vertical downward motion along fast fibers returns the trajectory to a point on P(L^+^) on the bottom sheet, completing the cycle.

When the singular periodic orbit reaches L^- ^it jumps up to a point on P(L^-^). If this point on P(L^-^) is in the singular funnel, then the orbit will move through the FN. Otherwise it will not. Let *δ *denote the distance measured along P(L^-^) from the phase point on P(L^-^) of the singular periodic orbit to the strong canard (SC in Figure 3). When the phase point is on the strong canard, *δ *= 0. Let *δ *> 0 when the phase point is in the singular funnel and *δ *< 0 when the phase point is outside the singular funnel. Singular canards are produced when *δ *> 0.

In Figure 3(a) the singular periodic orbit jumps to a point on P(L^-^) outside of the singular funnel (*δ *< 0), so it does not enter the FN. This orbit is a relaxation oscillation [31]. In Figure 3(b)*δ *> 0, so the orbit is a singular canard. Away from the singular limit, this singular canard perturbs to an actual canard that is characterized by small oscillations about L^+ ^[22]. The combination of these small oscillations with the large oscillations that occur due to jumps between upper and lower sheets yields mixed mode oscillations [24,32]. The small oscillations have zero amplitude in the singular case, which grows as $\sqrt{C_{m}}$ for *C_m _*sufficiently small [23]. A discriminating condition between relaxation and mixed mode oscillations is *δ *= 0, where the singular periodic orbit jumps to P(L^-^) on the SC curve.

**Figure 4:**
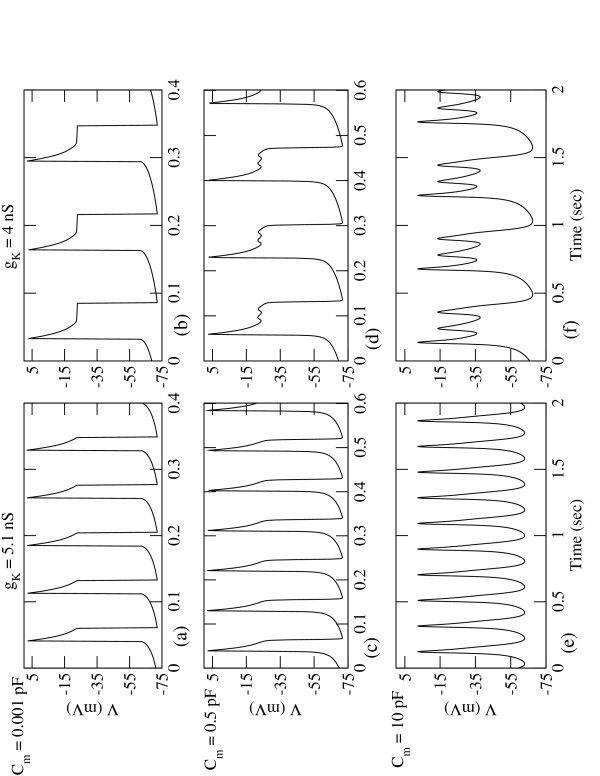
**Nearly-singular periodic orbits perturb to continuous spiking or mixed mode oscillations**. In both cases *g_BK _*= 0.4 nS, and *g_K _*= 5.1 nS in the left column, *g_K _*= 4 nS in the right column. *C_m _*is increased from top row to bottom row. (a), (c), (e) The singular periodic orbit does not enter the singular funnel (*δ *< 0) so it perturbs to continuous spiking. (b), (d), (f) The singular periodic orbit enters the singular funnel (*δ *> 0) so it perturbs to mixed mode oscillations or pseudo plateau bursting.

When *C_m _*> 0 the full system (Eqs.(2.1)-(2.3)) produces spiking for *δ *< 0 and mixed mode oscillations for *δ *> 0. Figure 4 shows these two different cases for *g_BK _*= 0.4 nS. For *g_K _*= 5.1 nS (*δ *< 0 in Figure 3(a)), the nearly-singular periodic orbit produced when *C_m _*= 0.001 pF (Figure 4(a)) perturbs to continuous spiking when *C_m _*= 10 pF (Figure 4(e)). When *g_K _*= 4 nS the singular periodic orbit enters the singular funnel (Figure 3(b)), so when *C_m _*is increased the singular orbit transforms to mixed mode oscillations. For *C_m _*= 0.5 pF mixed mode oscillations with small spikes are produced (Figure 4(d)). As *C_m _*is increased to 10 pF, mixed mode oscillations with larger spikes are produced. This is the genesis of pseudo-plateau bursting (Figure 4(f)).

## 4 Analysis of the desingularized system and folded nodes

**Figure 5:**
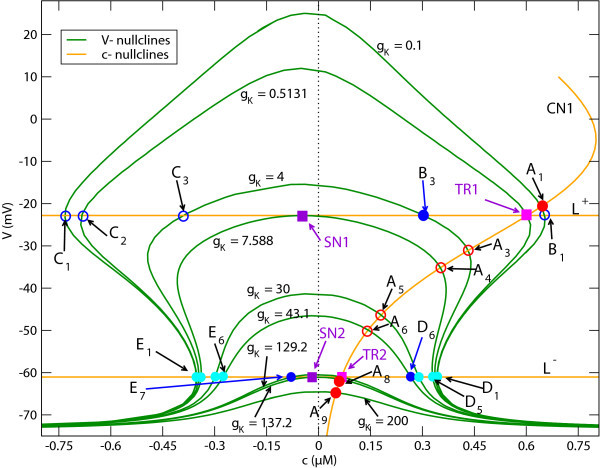
***V*-nullclines (green), the three-branched *c*-nullcline (orange), and singularities for *g_BK _*= 0.4 nS and different values of *g_K _*(units in nS)**. Filled circles represent stable singularities and unfilled circles represent unstable singularities. Red circles (filled or unfilled) are ordinary singularities. Filled and unfilled circles in blue are folded nodes and folded saddles, respectively. Filled circles in cyan are folded foci. The points TR1 and TR2 are transcritical bifurcations (type II folded saddle-node bifurcations) and SN1 and SN2 are standard saddle-node bifurcations (type I folded saddle-node bifurcations).

We next discuss the singularities of the desingularized system for a range of *g_K _*and *g_BK _*values (Figure 5). The system (with *g_BK _*= 0.4 nS) has a single-branched *V*-nullcline (green curve) that satisfies *F*(*V*, *c*, *n*) = 0 and a three-branched *c*-nullcline (orange curves) L^-^, L^+ ^and CN1. The curves L^-^, L^+ ^satisfy $\frac{\partial f}{\partial V}=0$, and are the same as the fold curves in Figure 3. The curve CN1 satisfies *αI_Ca _*+ *k_c _*= 0. There are folded singularities that are located at intersections of the *V*-nullcline with L^- ^or L^+^, and ordinary singularities that are located at intersections with CN1. For fixed *g_BK_*, changing *g_K _*affects the position of the *V*-nullcline but not the *c*-nullcline.

For values *g_K _*< 0.5131 nS, there is a stable node on CN1 (A_1_), which would be on the top sheet of the critical manifold. There are also two folded saddles on L^+ ^(B_1 _and C_1_) and two folded foci on L^- ^(D_1 _and E_1_). When *g_K _*is increased to 0.5131 nS the stable node A_1 _moves down and to the left and the folded saddle B_1 _moves to the left. These two equilibria coalesce at a transcritical bifurcation (TR1). This transcritical bifurcation corresponds to a bifurcation of folded singularities called a type II folded saddle-node [22,30,33]. Following this bifurcation, the folded singularity is a folded node. For *g_K _*= 4 nS, the equilibria on L^+ ^are the folded node (B_3_) and the folded saddle (C_3_). The equilibrium on CN1 (A_3_) is now a saddle point. There is no qualitative change of equilibria on L^-^.

When *g_K _*is increased to 7.588 nS the equilibria B_3 _and C_3 _coalesce at a saddle-node bifurcation point (SN1). This is a standard saddle-node bifurcation of folded singularities and is called a type I folded saddle-node [22,30,33]. As *g_K _*is increased to 43.1 nS, the folded focus D_5 _moves to the left and changes to a folded node at D_6_. The saddle points on CN1 move downward and to the left as *g_K _*is increased. For *g_K _*= 129.2 nS, the saddle point A_6 _coalesces with the fold node D_6 _at a second transcritical bifurcation (TR2); again a type II folded saddle-node. Beyond this, the ordinary singularity (A_8_, A_9_) is stable and the folded singularity becomes a folded saddle. Moreover the folded focus E_6 _has become a folded node (E_7_). As *g_K _*is increased further to 137.2 nS, there is a second type I saddle-node bifurcation (SN2) at which the folded node and the folded saddle coalesce and disappear. For the values *g_K _*> 137.2 nS, the only equilibrium is on CN1 and is an ordinary stable node (A_9_). This is on the bottom sheet of the critical manifold.

**Figure 6:**
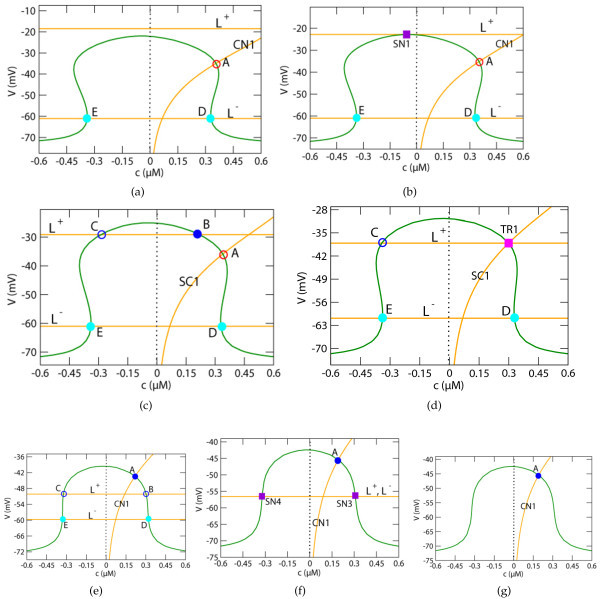
***V*-nullclines (green), *c*-nullclines (orange) and ordinary and folded singularities for a range of *g_BK _*values with *g_K _*= 7.588 nS**. (a) *g_BK _*= 0.2 nS, (b) *g_BK _*= 0.4 nS, (c) *g_BK _*= 1 nS, (d) *g_BK _*= 3.96 nS, (e) *g_BK _*= 20 nS, (f) *g_BK _*= 32.1224 nS, and (g) *g_BK _*= 32.2 nS. The color convention for equilibria is as in Fig. 5.

Varying *g_BK _*slightly affects the *V*-nullcline and strongly affects the *c*-nullcline in the (*c*, *V*)-phase plane. Increasing *g_BK _*moves the fold curves together, eventually taking the fold out of the critical manifold. Figure 6 shows qualitative changes in the equilibria when *g_BK _*is varied, with *g_K _*= 7.588 nS. When *g_BK _*= 0.2 nS there is a saddle point on CN1 (A) and two folded foci (D and E) on L^- ^(Figure 6(a)). When *g_BK _*is increased to 0.4 nS, the curve L^+ ^moves down and a type I folded saddle-node bifurcation occurs (SN1 in Figure 6(b)). When *g_BK _*is increased further, the saddle-node splits into a folded node (B) and a folded saddle (C) on L^+^, as shown for *g_BK _*= 1 nS in Figure 6(c).

The folded node (B) and the saddle point (A) coalesce at a transcritical bifurcation (type II folded saddle-node) when *g_BK _*= 3.96 nS (TR1 in Figure 6(d)). Beyond this, the ordinary singularity (A) is a stable node that lies on the top sheet of the critical manifold. When *g_BK _*= 20 nS the folded singularities are either saddles or foci, Figure 6(e). For *g_BK _*≈ 32.12 nS the two folded foci on L^-^change to folded nodes. Finally, when *g_BK _*is increased to 32.1224 nS, the fold curves L^+ ^and L^- ^merge. As a result, the folded saddles coalesce with the folded nodes at type I folded saddle-node bifurcations (SN3 and SN4 in Figure 6(f)). Beyond this, there is only a stable node (A in Figure 6(g)). The disappearance of the L^+ ^and L^- ^curves correspond to the disappearance of the fold in the critical manifold.

**Figure 7:**
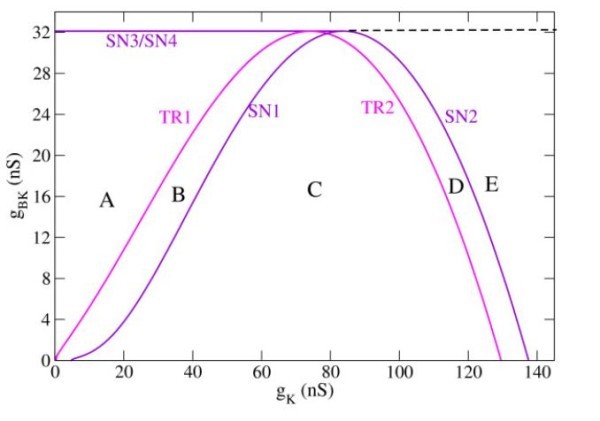
**Two-parameter bifurcation structure for the desingularized system**. The curves TR1 and TR2 represent the transcritical bifurcations (type II folded saddle-nodes). The curves SN1-SN4 represent saddle-node bifurcations (type I folded saddle-nodes). The horizontal line is where the fold curves L^+ ^and L^- ^coalesce. A codimension-2 bifurcation occurs at the intersection of the SN curves.

The two-parameter bifurcation diagram in Figure 7 summarizes the variations of the bifurcations in Figure 5 and Figure 6 over a range of *g_K _*and *g_BK _*values. The curves TR1 and TR2 correspond to the transcritical bifurcations (type II folded saddle-node bifurcations), and SN1-SN4 correspond to the saddle-node bifurcations (type I folded saddle-node bifurcations). At *g_BK _*= 32.1224 nS the L^+ ^and L^- ^lines coalesce into a single line. This contains the SN3 and SN4 bifurcations, up until SN3 and SN4 coalesce at a codimension-2 bifurcation (for *g_K _*= 83.7122 nS). For large *g_K_*, the L^+^/L^- ^line contains no folded singularities (dashed line).

For *g_K _*and *g_BK _*values in regions A, D and E there is only a stable node and the full system is in a depolarized (A) or hyperpolarized (D or E) steady state. In the left portion of region C there is a folded focus which becomes a folded node in the right portion of C. This family of folded singularities is on L^-^. In region D there is a folded node on L^- ^for negative values of *c*. Region B consists of the folded nodes on L^+^, and it is the key region for the existence of mixed mode oscillations, since *δ *> 0 for much of this region (shown below).

## 5 Twisted slow manifolds and secondary canards

The folded nodes discussed above are important since they yield small oscillations (for *C_m _*> 0) in all trajectories entering the singular funnel. In this section we explain the genesis of those oscillations (for more details, see [22,23,28,32]).

Folded nodes or saddles are characterized by the ratio of their eigenvalues. Let *λ*_1 _and *λ*_2 _be the eigenvalues of the folded singularity on the fold curve L^+ ^such that | *λ*_1_| < | *λ*_2_|. Define *μ *as

(5.1) $$\mu =\frac{\lambda_{1}}{\lambda_{2}}.$$

**Figure 8:**
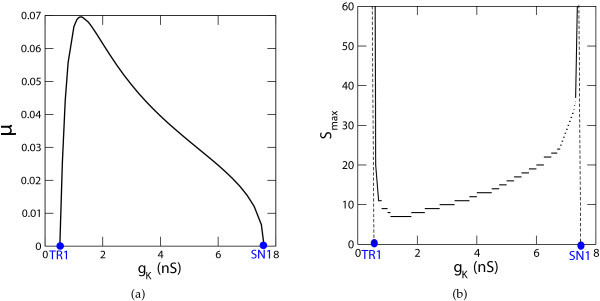
**The effects of varying *g_K _*on the eigenvalue ratio (*μ*) and the maximum number of oscillations (*S_max_*) for *g_BK _*= 0.4 nS**. (a) *μ *= 0 at TR1 and SN1. (b) *S_max _*is largest near the bifurcation points.

In region A of Figure 7, which consists of folded saddles, *μ *< 0. On the TR1 curve *μ *= 0 since *λ*_1 _= 0. Folded nodes occur in region B, so *μ *> 0. For *C_m _*> 0, but small, a trajectory approaching a folded node will oscillate, due to twists in the attracting and repelling sheets of the slow manifold. The maximum number of oscillations is given by [23,32]

(5.2) $$S_{max}=\left[\frac{\mu +1}{2\mu }\right],$$

which is the greatest integer less than or equal to $\frac{\mu +1}{2\mu }$. At a point in region B and close to the TR1 curve in Figure 7, *μ *> 0 but small. Hence, *S_max _*is large. Similarly on SN1 *μ *= 0, so in region B and close to the SN1 curve *μ *> 0 and small, so *S_max _*is large. Between these curves *μ *increases and *S_max _*declines. This is shown in Figure 8 for the case *g_BK _*= 0.4 pS. The small value of *μ *over the full range of *g_K _*values in (Figure 8a) suggests the system is close to a folded saddle-node bifurcation, either type I (SN1) or type II (TR1).

**Figure 9:**
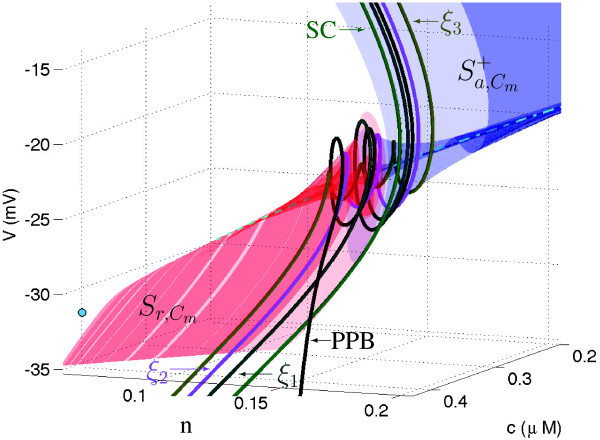
**A portion of the twisted slow manifold for *C_m _*= 2 pF, *g_K _*= 4 nS and *g_BK _*= 0.4 nS**. The top attracting (${\text{S}}_{a,C_{m}}^{+}$, blue surface) and middle repelling (${\text{S}}_{r,C_{m}}$, red surface) sheets of the slow manifold are twisted around the blue dashed curve, which is the axis of rotation. The primary strong canard (SC, green) moves from the attracting to the repelling sheet without any rotations. The secondary canards *ξ*_1 _(gray curve, one rotation), *ξ*_2 _(purple curve, two rotations) and *ξ*_3 _(gold curve, three rotations) flow from the attracting to repelling sheet with different numbers of rotations. A portion of the pseudo-plateau burst trajectory (PPB, black curve) is superimposed and has two small oscillations. The full system has unstable equilibrium (cyan, filled curcle).

The attracting sheets of the critical manifold (S_*a*_) and the repelling middle sheet (S_*r*_) come together at the fold curves L^+ ^and L^-^. For *C_m _*> 0, Fenichel theory [34] tells us that the critical manifold perturbs smoothly to invariant attracting $\left({\text{S}}_{a,C_{m}}\right)$ and repelling (${\text{S}}_{r,C_{m}}$) manifolds away from L^+ ^and L^-^. However, the critical manifold is non-hyperbolic on L^+ ^and L^-^, and perturbs to twisted sheets near these curves to preserve uniqueness of solutions [23,35]. Figure 9 shows how the top attracting ${\text{S}}_{a,C_{m}}^{+}$ (blue) and middle repelling ${\text{S}}_{r,C_{m}}$ (red) sheets of the slow manifold intersect and twist. The numerical method used to compute the slow manifolds was developed by Desroches et al. [36,37].

The primary weak canard corresponds to the weak eigendirection of the folded node. It is at the intersection of the invariant manifolds ${\text{S}}_{a,C_{m}}^{+}$ and ${\text{S}}_{r,C_{m}}$ and serves as their axis of rotation. All other canards twist about the primary weak canard; they follow ${\text{S}}_{a,C_{m}}^{+}$ as it twists and then follow ${\text{S}}_{r,C_{m}}$ for a distance as it twists. The primary strong canard, which corresponds to the strong eigendirection of the folded node, moves along ${\text{S}}_{a,C_{m}}^{+}$ to ${\text{S}}_{r,C_{m}}$ without any rotation (SC, green curve in Figure 9). Other, secondary, canards rotate a number of times, depending on how close they are to the primary strong canard. A secondary canard that makes k small rotations in the vicinity of the folded node is called the k*^th ^*secondary canard. Figure 9 shows the first (*ξ*_1_, gray), second (*ξ*_2_, purple) and third (*ξ*_3_, olive) secondary canards that make one, two and three rotations, respectively. For *C_m _*> 0, but small, there are *S_max _*- 1 secondary canards which divide the funnel region between the primary canards into *S_max _*subsectors [24]. The first subsector is bounded by the strong canard SC and the first secondary canard *ξ*_1 _and trajectories entering here have one rotation. The second subsector is bounded by *ξ*_1 _and *ξ*_2 _and trajectories entering here have two rotations. The last subsector is bounded by the last secondary canard and the primary weak canard. The maximal rotation number is achieved in the last subsector; trajectories entering here have *S_max _*rotations [23,28,32].

Figure 9 also shows a portion of the pseudo-plateau burst trajectory (PPB, black curve) for *C_m _*= 2 pF. It enters the funnel region in the rotational subsector bounded by *ξ*_1 _and *ξ*_2_, and hence, makes two full rotations and then leaves the repelling sheet as it moves towards the lower attracting manifold ${\text{S}}_{a,C_{m}}^{-}$. These rotations are the small oscillations or "spikes" during the burst active phase.

**Figure 10:**
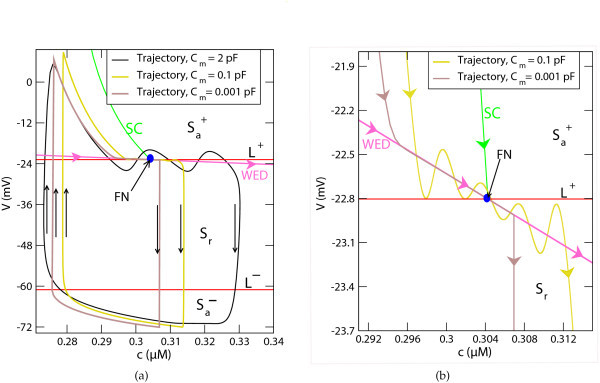
**(a) Pseudo-plateau burst trajectories are projected onto the (*c*, *V*)-plane for *g_K _*= 4 nS and *g_BK _*= 4 nS, and different values of *C_m_***. Key structures from the desingularized system are also shown. WED (pink curve) is the line tangent to the weak eigendirection of the folded node. (b) Magnification of panel (a) in the vicinity of the weak eigendirection and fold curve L^+^.

Figure 10(a) shows burst trajectories for three values of *C_m _*projected onto the (*c*, *V*)-plane. Also shown are L^+^, L^-^, the singular strong canard SC and the folded node of the desingularized system. Finally, the line along the weak eigendirection of the folded node is included (WED, pink curve). With *C_m _*= 0.001 pF the system is nearly singular and the "bursting" trajectory enters and leaves the folded node along the WED. The small oscillations near the folded node are too small to see. The region near the folded node is magnified in Figure 10(b). With *C_m _*= 0.1 pF the burst trajectory again passes through the folded node along the WED, but now the small oscillations are visible in Figure 10(b). The small oscillations of this burst trajectory first decrease and then increase in amplitude. This is often seen in mixed mode oscillations that are associated with a folded node singularity, in contrast to those associated with a singular Hopf bifurcation, where the amplitude of successive small oscillations increases [38]. Finally, with *C_m _*= 2 pF (the value used in Figure 9) the small oscillations are prominent even in the larger vertical scale used in Figure 10(a).

## 6 The boundaries of mixed mode oscillations

For a periodic mixed mode oscillation (i.e., pseudo-plateau bursting) solution to exist, there must be a folded node singularity and the periodic orbit must enter the funnel. In this section we construct curves in the two-parameter *g_K_*-*g_BK _*plane that form boundaries for the existence of mixed mode oscillations.

**Figure 11:**
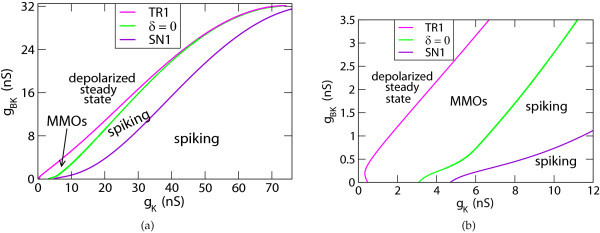
**(a) Mixed mode oscillation borders for *C_m _*→ 0**. The region of mixed mode oscillations (MMOs) is bounded by the two curves TR1 and *δ *= 0. Steady state and spiking solutions occur to the left of the TR1 curve and to the right of the *δ *= 0 curve, respectively. (b) Magnification of panel (a) for a smaller range of values of *g_K _*and *g_BK_*.

From Figure 7 we know that folded node singularities only occur in regions B and C (and in region D for negative values of the Ca^2+ ^concentration). Those in region C occur on L^- ^and the periodic orbit never enters the corresponding singular funnel. We therefore focus on region B. This region is highlighted in Figure 11(a). Above the TR1 curve the system has a depolarized stable steady state. Below the SN1 curve the system spikes continuously. Between these curves a folded node singularity exists, and the requirement for periodic mixed mode oscillations is that *δ *> 0. That is, the singular orbit must enter the singular funnel. Thus, the final curve delimiting the MMOs region is *δ *= 0 (the set of *g_K _*and *g_BK _*values at which the singular periodic orbit intersects both the strong canard and the curve P(L^-^)), shown in green in Figure 11. For parameter values between the *δ *= 0 and TR1 curves periodic mixed mode oscillations, i.e., pseudo-plateau bursting, exist and are stable. This critical region is magnified in Figure 11(b).

**Figure 12:**
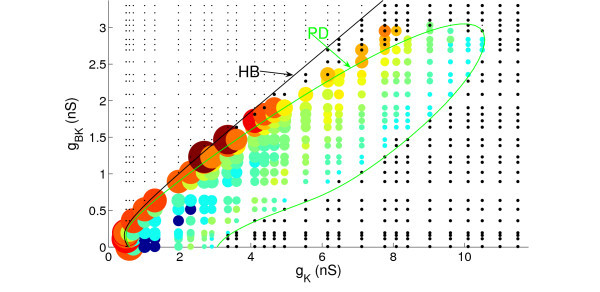
**The active phase duration and the number of spikes per burst of the full system for *C_m _*= 5 pF**. The system displays steady state, spiking or bursting solutions. Steady state and spiking solutions are represented by black dots and small black circles, respectively. The bursting region is bounded by the supercritical Hopf bifurcation (HB, black) and the right branch of the period doubling (PD, green) curves. The bursting patterns in this region are represented by colored circles. The size of a circle represents the active phase duration, with larger circles corresponding to longer active phase durations. The color of a circle represents the number of spikes per burst. Cyan circles correspond to smaller number of spikes per burst (minimum of two spikes) and the largest dark red circle corresponds to the largest number of spike per burst (36 spikes in a burst).

Figure 12 shows how the burst duration and the number of spikes in a burst vary over a range of *g_K _*and *g_BK _*values for *C_m _*= 5 pF. A similar map of parameter space was used previously in the analysis of a parabolic burster [39]. Two-parameter bifurcation curves of the full system (Eqs. (2.1)-(2.3)) are also shown. These include a curve of supercritical Hopf bifurcations (HB, black) and a curve of period doublings (PD, green). To the left of the HB curve the system is at a steady state (black dots), and to the right of and above the PD curve the system produces continues spiking (small black circles). For the values of *g_K _*and *g_BK _*inside the PD curve the system produces pseudo-plateau bursting oscillations (MMOs), represented by colored circles.

In the bursting region the active phase duration and the number of spike per burst vary with respect to the values of *g_K _*and *g_BK_*. The size of each circle represents the active phase duration, and the color of the circle (from cyan to dark red) represents the number of spikes in a burst. A burst with larger number of spikes has longer active phase duration, and in an actual cell this determines the amount of Ca^2+ ^influx and hormone released. The bursts that have the shorter active phase duration and the smaller number of spikes occur near the right branch of the PD curve. These bursts are represented by smaller cyan circles in Figure 12. For example, when *g_BK _*= 1 nS and *g_K _*= 6 nS the system produces bursting oscillations with three spikes per burst (as in Figure 4(f)). When one moves away from the right to the left branch of the PD curve by increasing *g_BK _*or decreasing *g_K _*the burst duration becomes longer and the number of spikes in a burst becomes larger. The longest active phase duration is about 8.4 sec and the largest number of spikes per burst is about 36, represented by the largest dark red circle. These values will change when *C_m _*is changed.

The region between the HB and the left branch of the PD curves is bistable between bursting and continuous spiking. Orange circles with small black circles at the centers represent bistable solutions that are simulated by varying the initial conditions. This shows that the borders of the bursting region are the HB and the right branch of the PD curves. The dark blue circles represent bursting oscillations without small oscillations since the amplitudes of the spikes are almost zero, i.e., the small oscillations are too small to see.

The results that are shown in Figure 12 are very consistent with the analysis of the mixed mode oscillations in Figure 11. The HB and TR1 curves overlap, demonstrating that for small *C_m _*the HB of the full system corresponds to a type II saddle-node bifurcation of the desingularized system. Also, the HB curve and the left branch of the PD curve are almost indistinguishable for small *C_m_*. For these *C_m _*values (*C_m _*< 0.001 pF), the right branch of the PD curve converges to the *δ *= 0 curve of the desingularized system. Hence, the left and right borders of the MMOs in the singular limit *C_m _*→ 0 pF correspond to the left and right borders of the bursting region of the full system for *C_m _*> 0, with the exception that the bursting region is smaller for larger values of *C_m_*. Also, the bistable region between the PD and HB curves only exists as the left PD moves away from the HB, which occurs as *C_m _*is increased.

In Figure 11 the MMOs region delimited by the TR1 and *δ *= 0 curves can be divided into subregions that have different numbers of small oscillations. For parameter values in the subregion near the curve *δ *= 0 the periodic orbit enters the funnel region near the strong (primary) canard. This subregion corresponds to the first subsector of the funnel region, and for *C_m _*> 0 only one small oscillation occurs in a burst. This corresponds to the jump from the lower attracting sheet to the upper attracting sheet and is not due to the folded node. When one moves leftward by decreasing *g_K_*, *δ *increases and the periodic orbit enters the funnel region through other subsectors. As a result, the number of small oscillations in a burst increases. When one moves to the subregion near or on the TR1 curve by decreasing *g_K _*further, the periodic orbit enters the funnel region through the last subsector. The number of small oscillations is closer to *S_max_*, the maximum number of spikes in a burst as determined by the eigenvalues of the folded node. Moreover, increasing *g_BK _*has the same effect as decreasing *g_K_*. These trends in the number of small oscillations obtained from an analysis of the desingularized system [28] are expressed far from the singular limit as shown in Figure 12 where *C_m _*= 5 pF. Here the longest bursts occur near the HB curves, as predicted.

## 7 A comparison with a two-fast/one-slow variable analysis

Using a one-fast/two-slow variable analysis we have shown the genesis of the spikes in a burst and how the number of spikes in a burst varies in the *g_K_*-*g_BK _*parameter space. The regions for steady states, pseudo-plateau bursting (mixed mode oscillations) and spiking are clearly identified in this parameter space (Figure 11). This has been done by investigating the qualitative changes of the desingularized system when parameters *g_K _*(Figure 5) and *g_BK _*(Figure 6) are varied, which are summarized in Figure 7.

**Figure 13:**
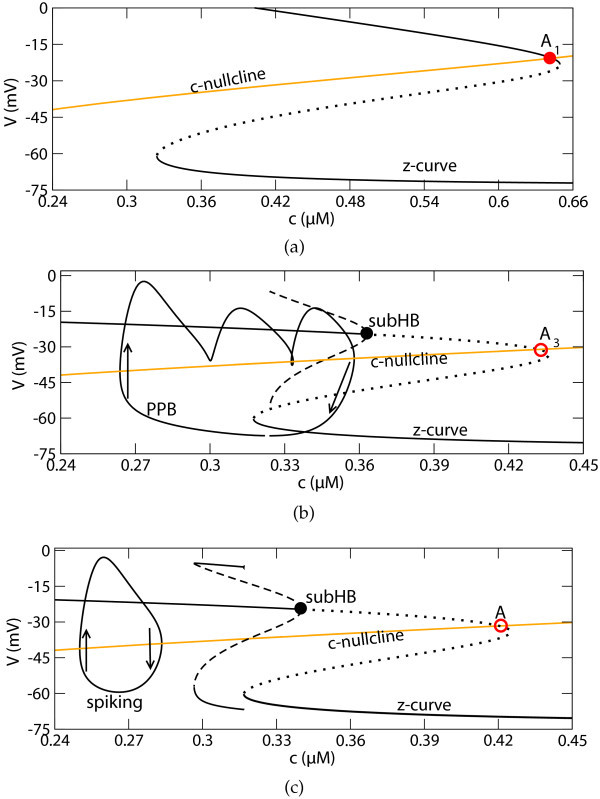
**Two-fast/one-slow analysis for *g_BK _*= 0.4 nS, *C_m _*= 10 pF and different values of *g_K_***. The black "z-curve" is the curve of equilibria of the *V*-*n *fast subsystem. This has stable (solid) and unstable (dashed) branches. (a) *g_K _*= 0.1 nS, the full system with stable equilibrium (A_1_) is in a depolarized steady state. (b) *g_K _*= 4 nS, the fast subsystem has an unstable limit cycle that emerges from the subcritical Hopf bifurcation (subHB). Pseudo-plateau bursting (PPB, black trajectory) is produced. The equilibrium of the full system (A_3_) is unstable. (c) *g_K _*= 5.1 nS, the full system produces periodic spiking that appears unrelated to the fast subsystem bifurcation structure. The equilibrium point of the full system (A) is unstable. In all panels the system is bistable over a range of *c*-values. The points A_1 _and A_3 _are the same equilibrium points as A_1 _and A_3 _in Fig. 5, respectively.

Here we investigate whether this information can be obtained from a standard two-fast/one-slow variable analysis. Figure 13(a) shows a bifurcation diagram of the *V*-*n *fast subsystem with *c *treated as a parameter (referred to as a "z-curve"). The subsystem is bistable over a large range of *c *values, with stable depolarized and hyperpolarized steady states, separated by saddle points. The *c*-nullcline is superimposed, now thinking of *c *as a slowly-changing variable rather than as a parameter. This is the standard approach used in a two-fast/one-slow variable analysis. In all three panels of Figure 13 parameters are set at *g_BK _*= 0.4 nS, *C_m _*= 10 pF, and *g_K _*is varied.

In Figure 13(a), with *g_K _*= 0.1 nS, there is an intersection of the *c*-nullcline on the upper stable branch at location A_1_. This is a stable equilibrium of the full 3-dimensional system, and corresponds to A_1 _in the analysis shown in Figure 5. Thus, both types of analysis indicate that the system will come to rest at a depolarized steady state when *g_K _*= 0.1 nS.

When *g_K _*is increased there is a subcritical Hopf bifurcation on the upper branch with emergent unstable periodic solutions of the fast-subsystem. This is shown in Figure 13(b) for the case *g_K _*= 4 nS. Pseudo-plateau bursting occurs for this and nearby values of *g_K_*. The full system unstable equilibrium (A_3_) corresponds to A_3 _in Figure 5.

The superimposed burst trajectory in Figure 13(b) only weakly follows the fast-subsystem bifurcation diagram. Most notably, there are no stable periodic solutions of the fast subsystem, only bistability between two steady states. Also, the trajectory never follows the lower branch of stationary solutions and greatly overshoots the lower knee.

The subcritical Hopf bifurcation migrates leftward when *g_K _*is increased to 5.1 nS. The unstable branch of periodics goes through a saddle-node bifurcation, yielding a branch of stable periodic solutions of the fast subsystem (Figure 13(c)). There is bistability between upper and lower branches of the z-curve which is typically a necessary condition for bursting with this type of analysis. However, bursting is not produced for this value of *g_K_*. Instead, the system spikes continuously.

This example illustrates that features well described by the one-fast/two-slow variable analysis are not at all well described by a standard two-fast/one-slow variable analysis. Most notably, the transition from bursting to spiking is well characterized in the one-fast/two-slow variable analysis as the point at which *δ *= 0. Note that this is not a bifurcation point of the desingularized system, but reflects the jump point from the lower sheet of the slow manifold to the the upper sheet. In contrast, the bursting to spiking transition is not predicted from the two-fast/one-slow analysis, and indeed the periodic spiking trajectory of the full system occurs over a range of the fast-subsystem bifurcation diagram that contains only stable equilibria. The one-fast/two-slow approximation is good even at higher values of *C_m_*, for example, when *C_m _*= 5 pF (Figure 12). Similar remarks apply for smaller values of *C_m_*, where the one-fast/two-slow approximation becomes more accurate while the two-fast/one-slow approximation does not. The two-fast/one-slow approximation becomes more accurate when *c *is much slower than both *V *and *n*, but in this case only a stable steady solution or a relaxation oscillation is produced.

## 8 Discussion

The canard mechanism has been used to understand mixed mode oscillations in several neuronal models [30,37,40-44]. In these examples, the small oscillations correspond to subthreshold oscillations that occur between the electrical impulses. We have previously analyzed pseudo-plateau bursting in a pituitary lactotroph model using canard theory [28]. However, the model used was a simplification in which the cytosolic free Ca^2+ ^concentration was treated as a fixed parameter and the second slow variable (in addition to the variable *n *used here) was an inactivation variable for an A-type K^+ ^current. In the current paper, we again focused on pseudo-plateau bursting in a pituitary lactotroph model, but now with emphasis on a BK-type K^+ ^current. In this analysis, we have examined the effects of changing the parameters *C_m_*, *g_K _*and *g_BK_*. The parameter *g_BK _*is important for producing bursting oscillations in actual pituitary cells in which bursting is converted to spiking when BK-type K^+ ^channels are blocked [45].

Here, using *C_m _*to control the separation in time scales, we identified two slow variables (*n*, *c*) and one fast variable (*V*). Using the one-fast/two-slow variable analysis we showed that pseudo-plateau bursting is a canard-induced mixed mode oscillation. There are two main requirements for the existence of these bursting oscillations [22-24,32]. One is that the desingularized system must have a folded node singularity, i.e., the eigenvalue ratio (*μ*) has to be positive. The second requirement is that the singular periodic orbit should enter the singular funnel and pass through the folded node, i.e., *δ *should be positive. In short, canard-induced mixed mode oscillations exist if both *μ *and *δ *are positive.

Using this technique we can understand several features of the burst and several trends that occur as parameters are varied. When both *μ *and *δ *are positive, small oscillations are produced during the active phase of a burst and their amplitude is proportional to $\sqrt{C_{m}}$ for *C_m _*sufficiently small [23]. We obtained the bursting borders in the (*g_K_*, *g_BK_*)-plane (Figure 11 and 12), and predicted how the active phase duration and the number of spikes per burst vary with changes in parameters.

The singular perturbation analysis performed here is technically more effective and informative in the singular limit (i.e., for sufficiently small values of *C_m_*) [22,23]. However, it provides useful information even far from this limit, as we showed in Figures 11 and 12. Eventually, as the singular parameter (*C_m_*) is increased sufficiently, new dynamics will be introduced, and the insights from the singular analysis are no longer valid.

The one-fast/two-slow decomposition used here contrasts with the two-fast/one-slow variable analysis used previously for pseudo-plateau bursting [10-13]. Our analysis explains the origin of the small-amplitude spikes that occur during the active phase of pseudo-plateau bursting, the transition between spiking and bursting, and information about how the number of spikes per burst varies with parameters. While the two-fast/one-slow variable analysis provides little information on these things, it does provide valuable information about how one can make a transition between plateau and pseudo-plateau bursting as one or more parameters are changed [12]. It also provides information about complex phase resetting properties [11] and the termination of spikes in a burst [17]. Both fast/slow decompositions are approximations, however, to a system that evolves on three time scales. Some studies [13,17,18] focus on the dynamics of the full system, and illustrate the complexity of the seemingly simple set of equations. The advantage of obtaining useful information of the full system by a two-fast/one-slow or one-fast/two-slow decomposition points to the fact that system (2.1)-(2.3) actually evolves on three time scales: *V *fast, *n *intermediate and *c *slow. This can also be seen by the magnitude of *μ *which is bounded from above by *μ_max _*≈ 0.07 (Figure 8(a)). Hence, we are close to folded saddle-node regimes (type I and type II) [33,38] and a more detailed bifurcation analysis may explain the relation between the two-fast/one-slow and one-fast/two-slow splitting. This is left for future work.

## Competing interests

The authors declare that they have no competing interests.

## Authors' contributions

WT, JT, TV, MW, and RB performed the analysis. WT wrote the manuscript with assistance from JT, TV, MW, and RB. All authors read and approved the final manuscript.
